# Genome-wide CRISPR/Cas9 library screen identifies C16orf62 as a host dependency factor for porcine deltacoronavirus infection

**DOI:** 10.1080/22221751.2024.2400559

**Published:** 2024-09-02

**Authors:** Ningning Ma, Mengjia Zhang, Jiaru Zhou, Changsheng Jiang, Ahmed H. Ghonaim, Yumei Sun, Pei Zhou, Guanghao Guo, Anouk Evers, Hongmei Zhu, Qigai He, Robert Jan Lebbink, Berend Jan Bosch, Wentao Li

**Affiliations:** a National Key Laboratory of Agricultural Microbiology, Hubei Hongshan Laboratory, College of Veterinary Medicine, Huazhong Agricultural University, Wuhan, People’s Republic of China; b Anhui Provincial Key Laboratory of Animal Nutritional Regulation and Health, College of Animal Science, Anhui Science and Technology University, Fengyang, People’s Republic of China; c Desert Research Center, Cairo, Egypt; d Department of Medical Microbiology, University Medical Center Utrecht, Utrecht, The Netherlands; e Virology Division, Department of Infectious Diseases & Immunology, Faculty of Veterinary Medicine, Utrecht University, Utrecht, The Netherlands

**Keywords:** Porcine deltacoronavirus, CRISPR/Cas9, C16orf62, aminopeptidase N, host factor

## Abstract

Porcine deltacoronavirus (PDCoV) is an emerging pathogen that can cause severe diarrhoea and high mortality in suckling piglets. Moreover, evidence of PDCoV infection in humans has raised concerns regarding potential public health risks. To identify potential therapeutic targets for PDCoV, we performed a genome-wide CRISPR/Cas9 library screening to find key host factors important to PDCoV infection. Several host genes in this screen were enriched, including ANPEP, which encodes the PDCoV receptor aminopeptidase N (APN). Furthermore, we discovered C16orf62, also known as the VPS35 endosomal protein sorting factor like (VPS35L), as an important host factor required for PDCoV infection. C16orf62 is an important component of the multiprotein retriever complex involved in protein recycling in the endosomal compartment and its gene knockout led to a remarkable decrease in the binding and internalization of PDCoV into host cells. While we did not find evidence for direct interaction between C16orf62 and the viral s (spike) protein, C16orf62 gene knockout was shown to downregulate APN expression at the cell surface. This study marks the first instance of a genome-wide CRISPR/Cas9-based screen tailored for PDCoV, revealing C16orf62 as a host factor required for PDCoV replication. These insights may provide promising avenues for the development of antiviral drugs against PDCoV infection.

## Introduction

Coronaviruses are classified into four genera, namely *Alphacoronavirus*, *Betacoronavirus*, *Gammacoronavirus*, and *Deltacoronavirus*, based on their genomic characteristics and evolutionary relationships [1,2]. Porcine deltacoronavirus (PDCoV), a member of the genus *Deltacoronavirus*, causes acute diarrhoea, vomiting, dehydration, and mortality in neonatal piglets [3]. PDCoV, also referred to as HKU15, was first reported in pigs in Hong Kong in 2012 [4,5]. Since then, it has been widely detected as an enteropathogenic coronavirus in swine populations across East/Southeast Asia and North America since 2014 [6–9]. PDCoV has a single-stranded positive-sense RNA genome of approximately 25.4 kb in size [10,11]. The genome encodes four structural proteins, including the spike (S) protein, envelope (E) protein, membrane (M) protein, and nucleocapsid (N) protein [12,13]. The S protein, which is localized on the virion surface, plays crucial roles in receptor binding as well as the fusion of viral and host cell membranes, thereby facilitating viral entry and infection [12,14,15].

PDCoV utilizes porcine APN as its protein receptor for cell entry [16], and it can also functionally employ APN orthologues of a wide range of species, including humans, pigs and cats [17], indicating a broad host range potential. Indeed, a wide range of animal species can be experimentally infected with the virus such as pigs, cattle, chicks, turkeys, and mice [18–21]. Recently, PDCoV infections in humans have also been reported, causing acute febrile illness in children, raising concerns about its zoonotic potential [22]. While APN knockout from cells abrogates PDCoV infection by approximately 90%, a fraction of cells remain susceptible to PDCoV, suggesting that the virus’ entry and replication are not entirely dependent on APN [17]. This observation underscores the importance of elucidating additional host factors involved in PDCoV infection to gain comprehensive insight into its pathogenic mechanisms, which may in turn contribute to the development of host-directed antiviral strategies. Recent studies have indeed identified several host factors that play crucial roles in PDCoV invasion and replication. For instance, Fang et al. reported that the solute carrier family 35 member A1 (SLC35A1) is a host factor required for PDCoV infection, acting by regulating cell surface sialic acid (SA) [23]. Peng et al. found that PDCoV replication was significantly inhibited in TMEM41B knockout cells [24]. Zhang et al. showed that the host proteases cathepsin L (CTSL) and cathepsin B (CTSB) activate PDCoV entry through the endosome pathway [25]. Furthermore, Huang et al. demonstrated that the heat shock protein 90 alpha family class B member 1 (HSP90AB1) is a host factor that promotes PDCoV replication [7]. Hence, the host genes involved in PDCoV infection should be further determined to enrich our understanding of viral infection and pathogenic mechanisms.

In the present study, we selected cells resistant to PDCoV infection by using mutagenized Huh7 cells transduced with a genome-wide CRISPR/Cas9 library. Multiple rounds of PDCoV infection were performed, and resistant cells were selected based on cell survival. Several host genes were enriched in the surviving cells, including ANPEP, which encodes the PDCoV receptor APN, as well as C16orf62, not previously known to play a role in PDCoV infection. The dependency of PDCoV replication on C16orf62 was confirmed by *in vitro* experiments showing resistance to PDCoV replication in cell culture after C16orf62 knockout. We demonstrate that, although C16orf62 does not directly interact with the PDCoV S1 protein, the knockout of C16orf62 resulted in reduced APN expression at the cell surface and impaired virus adsorption. Our study shows the crucial role of C16orf62 for infection by PDCoV and highlights its potential as a target for the development of anti-PDCoV therapeutics.

## Materials and methods

### Plasmid construction

To construct the lentivirus single guide RNA (sgRNA) expression vector, we first digested the sgLenti (MP-783) [26] vector using the *Aar I* restriction enzyme. Then, we annealed the paired oligonucleotides corresponding to the sgRNAs and cloned them into the linearized vector. The recombinant plasmids pQCXIP-C16orf62-V5, pQCXIP-C16orf62-Flag, pQCXIP-APN-Flag, and pQCXIP-APN-HA were generated using MultiF Seamless Assembly (ABclonal, RK21020, China) by cloning the sequences into the pQCXIP-V5, pQCXIP-Flag, or pQCXIP-HA vector. All primer sequences are listed in Supplementary Table 1.

### Cell culture and transfection

Human hepatoma (Huh7) cells, Human embryonic kidney 293 T (HEK 293 T) cells, Pig kidney (LLC-PK1) cells, Swine testicular (ST) cells, Pig kidney (PK-15) cells, African green monkey kidney (Vero-CCL81) cells, and Human cervical cancer (HeLa-R19) cells were purchased from the Cell Bank of the Chinese Academy of Sciences (Shanghai, China). Cas9-expressing cell lines LLC-PK1-Cas9 and ST-Cas9 were generated through lenti-Cas9-Blast transduction are preserved in our laboratory. All cells were cultured in DMEM (Gibco, Grand Island, NY, USA) supplemented with 10% fetal bovine serum (ExCell Bio, Shanghai, China), 100 U/mL penicillin, and 100 μg/mL streptomycin (Gibco, Grand Island, NY, USA) and incubated at 37°C with 5% CO_2_. Prior to study beginning, all cell lines were tested and confirmed to be mycoplasma negative. Transfections were performed with jetPRIME® transfection reagent (Polyplus, Paris, France) according to the manufacturer’s instructions.

### Viruses

PDCoV was purchased from the USDA (USA) and then used for CRISPR/Cas9 screening. PDCoV-GFP was generated using the reverse genetic system as previously described [27]. The virus was propagated and titrated on LLC-PK1 cells (a pig kidney cell line known to be highly permissive to PDCoV infection) [28,29] in DMEM supplemented with 10 μg/mL TPCK-treated trypsin (4370285, Sigma). VSV-GFP was kindly provided by Prof. Qigai He, and was propagated in PK-15 cells. TGEV strain WH-1 (GenBank accession number: HQ462571.1) was isolated and has been preserved in our laboratory.

### Lentivirus production and transduction

To produce lentivirus, a co-transfection of 5 μg of the lentiviral vector, 0.5 μg of pMD2.G plasmid, and 5 μg of psPAX2 plasmid was performed in HEK 293 T cells per 100 mm dish using jetPRIME^®^ transfection reagent according to the manufacturer’s instructions. 72 h after transfection, the supernatants were filtered through a 0.45 μm low protein binding membrane (Millipore, USA), and then centrifuged at 153,700×*g* at 4°C for 2.5 h. The virus pellets were resuspended in phosphate buffered saline (PBS) and stored at −80°C until further use. Target cells were transduced with the lentiviruses in the presence of 8 μg/mL polybrene (Beyotime, China). After 20 h of transduction, the inoculum was replaced with fresh medium [30].

### Generation of the cas9-expressing cell line

Huh7 cells were transduced with pSicoR-SpCas9-ZeoR (RP-612) lentivirus. To allow screening using the genome-wide sgRNA library described below, the pSicoR-CRISPR-PuroR vector was altered to replace the PuroR for ZeoR and remove the U6 promoter. This vector drives expression of a human codon-optimized nuclear-localized streptococcus pyogenes Cas9 gene in the absence of a U6 promoter – sgRNA cassette [31]. On the third day after transduction, the medium was replaced with fresh growth medium containing 100 μg/mL Zeocin (Thermo Fisher Scientific, USA). The Zeocin-resistant cells were collected and reseeded into 100 mm dishes at a concentration of 100 cells per dish to generate single-cell clones [32]. Seven days later, the Cas9 expression in the single-cell clones was tested by immunofluorescence staining and flow cytometry. The resulting cell lines were designated as Huh7-Cas9.

### Genome-wide crispr/cas9 library screen

A genome-wide sgRNA library consisting of ±260,000 unique sgRNA sequences [26] was introduced into ∼100 million Huh7-Cas9 positive cells by means of lentiviral transduction. The final library coverage was >400 fold. Transduced cells were selected with 3 µg/mL puromycin (Sigma–Aldrich, USA) to ensure full selection of sgRNA-transduced cells. At 7 days post-transduction, >98% of the cells expressed the mCherry marker, which was present on the lentiviral sgRNA library vector. Prior to screening, we first examined PDCoV-induced cell death following infection with different multiplicities of infection (MOI) values of 0.1, 1.0, and 10. Additionally, cytopathic effects (CPE) were observed approximately 2 days after PDCoV infection. Based on these observations, we selected an MOI of 1 as the optimal titre for PDCoV-induced cell death in Huh7-Cas9 cells. At 21 days post transduction, ∼100 million sgRNA-positive cells were infected with PDCoV at an MOI of 1. At 96 h postinfection (hpi), the cells were washed with DMEM to remove dead cells. The remaining cells were cultured in DMEM supplemented with 10% FBS and 1% penicillin–streptomycin solution for subsequent rounds of PDCoV infection. After three rounds of infection, the surviving resistant cells were expanded and subjected to deep sequencing analysis as described previously [26].

### Generation of knockout cells

Each gene was targeted with guide RNA using sgRNA Designer (http://crispor.tefor.net/crispor.py). The primers corresponding to the guide RNAs were synthesized and cloned into the sgLenti (MP-783) lentiviral vector [26]. This was followed by lentiviral production and transduction to the LLC-PK1-Cas9 or ST-Cas9 cell line. Three days later, the cells were selected by puromycin (Sigma–Aldrich, USA) for 2 days (4 μg/mL for LLC-PK1-Cas9 cells, 3 μg/mL for ST-Cas9 cells). Next, control DNA (untreated cells) and DNA from CRISPR/Cas9-edited cells were amplified and subjected to sequencing analysis. It is important to note that due to the lack of a functional anti-candidate antibody, we could not perform a Western blot analysis to validate the expression of the target genes in these polyclonal cells. Clonal lines were generated by limited dilution and verified by sequencing of the genomic target region.

### Real-time reverse transcription PCR (qRT–PCR) analysis

Total RNA was extracted from cells using TransZol Up (Transgen Biotech, China), while viral RNA was extracted from cell suspensions using a Viral RNA Extraction Kit (TaKaRa Bio, Japan) following the manufacturer’s instructions. The concentration and quality of the extracted RNA were assessed using a NanoDrop 2000 spectrophotometer (Thermo Fisher Scientific, USA). cDNAs were synthesized using the HiScript III 1st Strand cDNA Synthesis Kit (Vazyme #R312, China). For quantification, real-time quantitative polymerase chain reaction (qRT–PCR) was performed using the cDNA products as templates. The qRT–PCR was carried out with *Premix Ex Taq*™ (TaKaRa Bio, Japan) following the manufacturer’s instructions. Briefly, the PCR mixture (25 µL) consisted of 12.5 µL of *Premix Ex Taq* (Probe qPCR) (2×), 0.5 µL of forward primer (10 µM), 0.5 µL of reverse primer (10 µM), 1 µL of Probe, 8.5 µL of ddH_2_O and 2 µL of cDNA template. The results were monitored using a CFX96 Real-Time PCR Detection System (Bio-Rad, USA) following the programme: one cycle of 30 s at 95°C, followed by 45 cycles of 5 s at 95°C and 30 s at 60°C. The PDCoV M protein coding sequence (GenBank accession number MF095123) was cloned into the pMD19-T vector and used as a standard for the quantification of PDCoV copy numbers. All primers used in quantitative PCR are listed in supplementary Table 1.

**Table 1.** Antibodies and proteins used in this study.

Antibody	Name	Supplier	Catalog no.
V5	Mouse anti V5-Tag mAb	ABclonal	AE017
APN	ANPEP Rabbit mAb	ABclonal	A11669
GAPDH	GAPDH Rabbit pAb	ABclonal	AC001
Flag	Mouse anti Flag-Tag mAb	They are generated and preserved in our laboratory	
TGEV S1	Mouse anti TGEV S1 antibody		
PDCoV S1	Human anti PDCoV S1 antibody		
Secondary antibody	HRP goat anti-human IgG (H + L)	Jackson ImmunoResearch	109-035-088
	HRP goat anti-rabbit IgG (H + L)	ABclonal	AS014
	HRP goat anti-mouse IgG (H + L)	ABclonal	AS003
	Alexa Fluor® 488 AffiniPure™ Donkey Anti-Human IgG (H + L)	Jackson ImmunoResearch	709-545-149
	Alexa Fluor® 594 AffiniPure™ Goat Anti-Human IgG, F(ab’) ₂ fragment specific	Jackson ImmunoResearch	109-585-006
Protein	PDCoV S1-hFc	They are generated and preserved in our laboratory	
	TGEV S1-mFC		
	IgG-hFc		

### Cell viability assays

Cells were seeded into 96-well plates (5 × 10^3^ cells per well). 10 μL of CCK8 (Beyotime, China) was added to the cells, and the optical density (OD) value of the cells was measured at 450 nm using a spectrophotometer (Thermo Fisher Scientific, USA). To ensure the reliability of the results, three independent experiments were performed, and each experiment included five replicates (quintuplicates) for each condition or treatment.

### Virus titration

LLC-PK1 wild-type (WT) and C16orf62 knockout cells were seeded into 24 well plates and infected with PDCoV at an MOI of 0.1. The cell supernatants were harvested at 6, 12, 24, 36, 48, and 60 hpi. Next, LLC-PK1 cells were seeded in 96-well plates and were infected with 10-fold serial dilutions of virus samples in eight replicates. The cells were observed at 48 h after infection, and TCID_50_ was calculated using the Reed–Muench method.

### Generation of the c16orf62-overexpressing cells

To construct the C16orf62-overexpressing cells, the coding sequences of C16orf62 were cloned into the pQCXIP-V5 vector. To rescue C16orf62 expression, PAM sequences flanking binding sites of sgRNA and the sgRNA target sequences were mutagenized in the coding sequence of C16orf62, by introducing silent mutations. All primer sequences are listed in supplementary Table 1. The resulting vector was used to generate lentiviral particles. LLC-PK1, ST, Vero-CCL81, and HeLa-R19 cells were transduced with pQCXIP-C16orf62-V5 lentivirus. After 20 h transduction, the supernatant was removed, and the medium was replaced with fresh medium. Following an additional 48 h, the transduced cells were selected by culture with puromycin (Sigma–Aldrich, USA) for 2 days (4 μg/mL for LLC-PK1 cells, 3 μg/mL for ST cells, 6 μg/mL for Vero-CCL81 cells, and 2 μg/mL for Hela-R19 cells) to enrich lentivirus transduced cells. The overexpression of C16orf62 in the cells was confirmed by Western blot with anti-V5-antibody (ABclonal, AE017, China).

### Western blot assay

For Western blot, cellular proteins were extracted using a total protein extraction kit (Beyotime, China) according to the manufacturer’s instructions. The protein samples were then separated by 10% sodium dodecyl sulphate (SDS)-polyacrylamide gel electrophoresis (PAGE) and then transferred onto polyvinylidene difluoride (PVDF) membranes (Millipore, USA). PVDF membranes were blocked with 5% nonfat milk at room temperature for 2 h and then incubated overnight at 4℃ with the specific primary antibodies. Subsequently, the membranes were washed to remove unbound antibodies and incubated with horseradish peroxidase (HRP)-conjugated anti-human, mouse or rabbit antibodies for 1 h at room temperature and visualized using an Omni-ECL™ Femto Light Chemiluminescence Kit (Epizyme Biotech, China). Blots were visualized using a ChemiDoc MP Imaging System (Bio-Rad, USA). The antibodies used in this study are listed in Table 1.

### Flow cytometric quantification of PDCoV infected cells

The cells were detached from the tissue culture plates with trypsin-EDTA (Gibco, USA), centrifuged at 300×*g* for 3 min and washed twice with PBS. Then, cells were fixed with 4% paraformaldehyde at room temperature for 15 min, and the paraformaldehyde was removed by washing the cells twice with PBS. The percentage of GFP-positive cells was measured by flow cytometry (Cytoflex-LX, USA). All data were analysed with FlowJo V10 (FlowJo, Ashland, USA).

### Indirect immunofluorescence assay

For indirect immunofluorescence assay (IFA), cells were washed twice with PBS and fixed with 4% paraformaldehyde for 15 min at room temperature. Then, cells were washed three times with PBS and permeabilized with 0.1% Triton X-100 for 15 min at room temperature, blocked in 5% bovine serum albumin (BSA) in PBS for 1 h at room temperature, then incubated with primary antibody overnight at 4°C. The primary antibodies were detected using Alexa 488 or 594-labeled anti-human or anti-mouse antibodies. To visualize cell nuclei, 4′,6-diamidino-2-phenylindole (DAPI) (Sigma, USA) was added at room temperature for 7 min in the dark for counterstaining. Cell observation and imaging were performed using a fluorescence microscope (EVOS^®^ FL, Thermo Fisher Scientific, USA).

### PDCoV binding and internalization assay

Binding and internalization assays were performed as described previously with some modifications [33]. To determine PDCoV binding, LLC-PK1 and LLC-PK1 C16orf62 knockout cells were incubated with PDCoV at 10 or 50 MOI at 4°C for 1 h. The supernatant was then removed, and the cells were washed three times with ice-cold PBS to remove unbound virus. For the internalization assay, cells were incubated with PDCoV at 1 or 2 MOI, after binding at 4℃ for 1 h, cells were transferred to 37℃ for 1 h to allow virus entry and washed twice with acidic PBS (pH = 1.3) at 4°C to remove noninternalized particles. Subsequently, the cell lysates were subjected to qRT–PCR analysis by quantifying the PDCoV M copy number.

### Co-immunoprecipitation

To validate the interaction between PDCoV S1 and APN, 1 × 10^7^ HEK 293 T cells were seeded in 10-cm cell culture dishes and transfected with pQCXIP-APN-HA for 36 h. To validate the interaction between PDCoV S1 and C16orf62, 1 × 10^7^ ST cells overexpressing C16orf62 were seeded in 10-cm cell culture dishes. The cells were lysed using lysis buffer (Beyotime, China) at 4°C for 15 min. After sonication on ice, the cell lysates were centrifuged at 14,000×*g* for 10 min, and the supernatants were incubated with protein A-conjugated agarose beads at 4°C for 1 h to precipitate human Fc-tagged proteins. PDCoV S1-Fc protein and IgG were incubated with protein A-conjugated agarose beads at 4°C for 4 h to precipitate Fc-tagged PDCoV S1 protein or IgG. A portion of the supernatant from the lysed cells was used in the whole-cell extract assay. The remaining supernatant was immunoprecipitated with Fc-tagged PDCoV S1 protein or IgG overnight at 4°C. The beads were washed five times with ice-cold lysis buffer, boiled for 10 min in SDS-PAGE loading buffer, and then subjected to Western blot analysis. To validate the interaction between C16orf62 and APN, the lysate supernatants containing APN-HA and C16orf62-V5 were incubated for 4 h with gentle rocking at 4℃ and then incubated overnight with mouse mAb against V5 tag with gentle rocking at 4℃. Protein A/G beads washed with cell lysate were added to the supernatants and incubated with gentle rocking for 4 h at 4℃. The beads were washed five times with ice-cold lysis buffer, boiled for 10 min in SDS-PAGE loading buffer, and then subjected to Western blot analysis.

### Confocal microscopy

Cellular co-localization of C16orf62 and APN was analysed using confocal immunomicroscopy. HEK 293 T cells were transfected with either pQCXIP-C16orf62-V5 or pQCXIP-APN-Flag vectors alone, or co-transfected with both vectors. At 24 h post transfection, cells were washed three times with PBS and permeabilized with 0.1% Triton X-100 for 15 min at room temperature, blocked in 5% BSA in PBS for 1 h at room temperature, then incubated with primary antibody overnight at 4°C. The primary antibodies were detected using Alexa 488 or 594-labeled anti-human or anti-mouse antibodies. To visualize cell nuclei, DAPI was added at room temperature for 7 min in the dark for counterstaining. Finally, the images were captured using an Olympus FV1000 confocal microscope. The antibodies used in this study are listed in Table 1.

### TGEV/PDCoV S1 protein cell surface staining

Three μg/well of pQCXIP-C16orf62-Flag or pQCXIP-APN-Flag vectors were transfected into Vero-CCL81 cells. At 36 h post transfection, the cells were fixed with 4% paraformaldehyde for 15 min at room temperature, washed three times with PBS, and incubated with 10 μg/mL of TGEV S1-Fc/PDCoV S1-Fc protein at 4°C for 2 h. The binding signal was detected using an Alexa 488 or 594 labelled anti-human antibody. To visualize cell nuclei, DAPI was added at room temperature for 7 min in the dark for counterstaining. Cell observation and imaging were performed using a fluorescence microscope (EVOS^®^ FL, Thermo Fisher Scientific, USA).

### Statistical analysis

All the data, excluding the genetic screening and single-cell sequencing data, were statistically analysed using GraphPad Prism software. A significance level of *p *< .05 was considered statistically significant (*), while *p *< .01 was regarded as highly significant (**), an even higher level of significance, *p *< .001, was designated as (***), *p *< .0001, was designated as (****), and “ns” indicates no significant difference.

## Result

### CRISPR/Cas9 library screen identifies host genes essential for PDCoV infection

**Figure 1.**
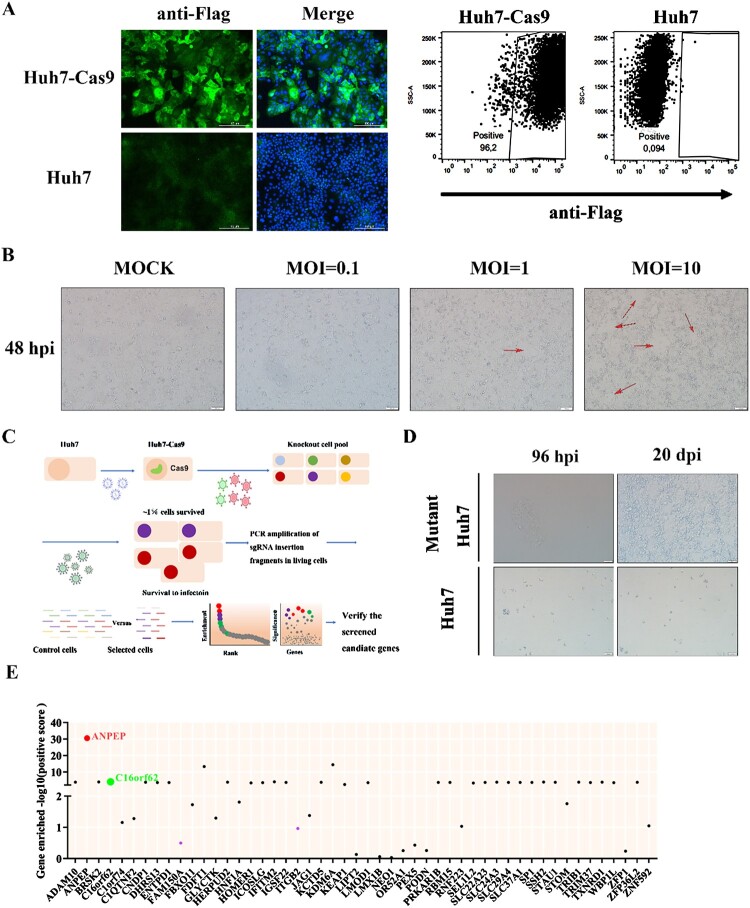
Genome-wide CRISPR/Cas9-based genetic screens in human cells unveiling host factors for PDCoV infection. (A) The left panel shows the expression of Cas9 protein in Huh7 cells as assessed by IFA using an anti-Flag monoclonal antibody. The right panel displays the expression of Cas9 protein in Huh7 cells as assessed by flow cytometry using an anti-Flag monoclonal antibody. Scale bar = 100 μm. (B) Determination of the optimal infection titre of PDCoV-induced Huh7 cells death. PDCoV-induced CPE is indicated by red arrows. Mock represents non-infected cells, used as a negative control. Scale bar = 100 μm. (C) Workflow and screening strategy for the CRISPR/Cas9 genetic screens in Huh7 cells. Huh7 cells stably expressing Cas9 were mutagenized by transduction with the lentiviral human sgRNA libraries, and cells were then repeatedly infected with PDCoV (MOI = 1). Cells surviving from the virus challenge were isolated, and their genomic DNA (gDNA) was extracted and sgRNA sequences were amplified by PCR and sequenced. (D) Cells survived from PDCoV infection in the mutant library cells group. At 21 days post transduction with the sgRNA library, ∼100 million sgRNA-positive cells were infected with PDCoV at an MOI of 1. At 96 hpi, the cells were washed with serum-free DMEM to remove dead cells. The remaining cells were cultured in DMEM supplemented with 10% FBS and 1% penicillin-streptomycin solution until day 20 post infection. Scale bar = 50 μm. (E) Enrichment scores of the top 50 genes in the libraries. The Y-axis represents the enrichment significance of gene knockouts compared with a non-selected control population.

Genome-wide CRISPR/Cas9 screens have enabled the identification of host factors required for efficient virus infection [34–37]. To identify host factors involved in PDCoV infection, we performed a genome-wide CRISPR/Cas9 library screen in PDCoV-susceptible Huh7 cells. First, we established a Huh7 cell line stably expressing Cas9 (Huh7-Cas9) (Figure 1(A)). Prior to screening, we examined PDCoV-induced cell death following infection at MOI of 0, 0.1, 1, and 10. We observed CPE for the different MOIs at approximately 2 days after PDCoV infection (Figure 1(B)). Here, we chose an optimal titre for PDCoV-induced cell death in Huh7-Cas9 cells with an MOI of 1. Huh7-Cas9 cells were mutagenized using a genome-wide sgRNA library. Mutagenized cells were challenged with three rounds of PDCoV infection, and parental Huh7-Cas9 cells were used as a negative control to confirm the cell death caused by PDCoV infection in each round (Figure 1(C and D)). Virus-resistant cells were collected and the abundance of sgRNAs in the untreated and virus-selected cell population was determined by Illumina sequencing, after which a gene enrichment analysis was performed. The ANPEP gene, which encodes the known receptor APN of PDCoV [17], was significantly enriched (Figure 1(E)), confirming the importance of this host factor in virus replication, and demonstrating the reliability of the screening. Besides ANPEP, several candidate genes including ADAM metallopeptidase domain 10 (ADAM10), BR serine/threonine kinase 2 (BRSK2), and C16orf62 as well as others not yet reported to be involved in PDCoV infection, were also enriched in the CRISPR/Cas9 screen (Figure 1(E)).

### Knockout of candidate host factor genes inhibits PDCoV infection

**Figure 2.**
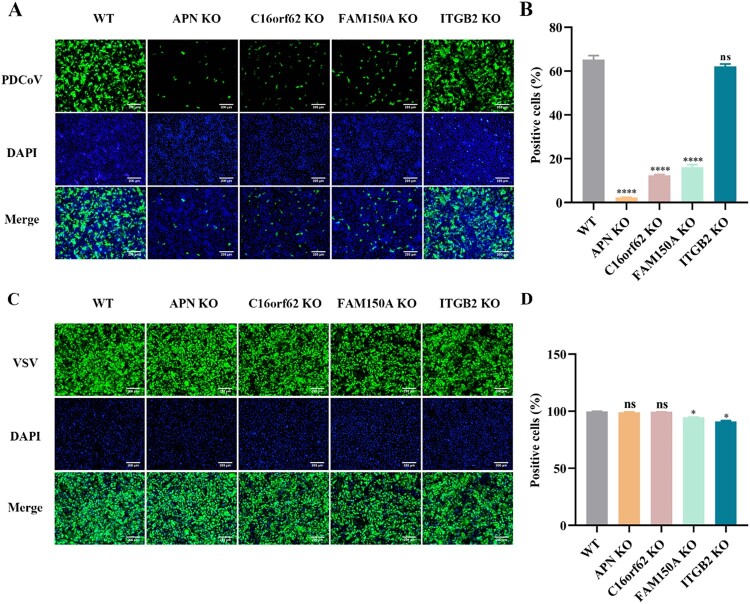
Validation of hits from genetic screens. (A) LLC-PK1 polyclonal knockout cells for APN, C16orf62, FAM150A and ITGB2 were infected with PDCoV-GFP (MOI = 0.1) for 24 h, after which infected (GFP-positive) cells were visualized by fluorescence microscopy upon staining of the cell nuclei with DAPI, or (B) quantified by flow cytometry analysis. (C) LLC-PK1 polyclonal knockout cells were infected with VSV-GFP (MOI = 0.001) for 24 h, after which infected (GFP-positive) cells were visualized by fluorescence microscopy upon staining of the cell nuclei with DAPI, or (D) quantified by flow cytometry analysis. Images were captured at 10× magnification. Scale bar = 200 μm. Error bars indicate the standard deviations of data from three independent experiments. *p < .05, ****p < .0001, and “ns” denotes no significant difference.

To assess the association of the top-ranking genes with PDCoV infection, four genes encoding proteins with plasma membrane localization, including ANPEP, C16orf62, ALK and LTK ligand 1 (ALKAL1/FAM150A) and integrin subunit beta 2 (ITGB2) were selected for further validation. For each of the four candidate genes, LLC-PK1 polyclonal knockout cells were constructed using the CRISPR/Cas9 gene editing system. These polyclonal cells included superimposed peaks in the sequencing chromatogram (Figures S1A and C), indicating the successful construction of candidate gene knockout polyclonal cells. Knockout of the C16orf62 and FAM150A genes inhibited PDCoV replication. However, the knockout of the ITGB2 gene did not confer resistance to the PDCoV infection (Figure 2(A and B)). To ensure that the observed effects were specific to PDCoV and not due to nonspecific effects on viral infection in general, LLC-PK1 polyclonal knockout cells were infected with GFP-labeled vesicular stomatitis virus (VSV), and no obvious effect was observed on VSV infection (Figure 2(C and D)). Moreover, knockout of these genes had no effect on cell viability (Figure S1B). Among the validated genes, C16orf62 was prioritized for further mechanistic studies due to its relatively strong ability to inhibit PDCoV replication and the lack of current reports on the role of C16orf62 in coronavirus infection.

### C16orf62 is required for PDCoV infection

To further study the role of C16orf62 in PDCoV infection, clonal C16orf62 knockout cell lines were generated. Next, sequencing confirmed that the C16orf62 knockout cell lines had more nucleotide deletions predicted to cause a frameshift mutation in the coding regions of the targeted gene (a noninteger multiple of 3) (Figure 3(A and B)). Compared to the parental LLC-PK1 cells, LLC-PK1 C16orf62 knockout cells infected with PDCoV exhibited a greatly reduced CPE (Figure 3(C)). Additionally, C16orf62 knockout cells were less susceptible to PDCoV-GFP infection as detected by fluorescent microscopy and flow cytometry. A similar effect was observed in the APN knockout cells (Figure 3(D and E)). Furthermore, PDCoV infection was significantly reduced in C16orf62 knockout cells as determined by qRT–PCR and TCID_50_ assays (Figure 3(F and G)). To investigate the impact of C16orf62 knockout on PDCoV infection in other susceptible cell lines, a C16orf62 knockout ST cell line was established, demonstrating robust resistance to PDCoV infection (Figure 3(H and I)). To exclude the possibility of other factors contributing to the inhibition of PDCoV infection in knockout cells, rescue experiments were conducted in ST C16orf62 knockout cells by transfection of a C16orf62 encoding plasmid (Figure S2). Parental ST cells, ST C16orf62 knockout cells, and C16orf62 – rescue were infected with PDCoV (MOI = 0.1), and the percentage of infected cells was assessed through immunostaining. Exogenous expression of C16orf62 in ST C16orf62 knockout cells partially restored PDCoV replication, compared to that in ST C16orf62 knockout cells (Figure 3(J and K)). Taken together, these results suggested that C16orf62 is involved in PDCoV infection. Figure 3.C16orf62 is a host factor required for PDCoV replication. (A) Establishment of the C16orf62 knockout LLC-PK1 cell line. The sequencing result showed that 7-base deletions was detected in Exon 3. (B) Establishment of the C16orf62 knockout ST cell line. The sequencing result showed that 38-base deletions was detected in Exon 1. The PAM site is marked in blue lettering. The red characters “-” indicate the deleted bases in the knockout cell lines. (C) LLC-PK1 C16orf62 knockout cell line and WT cells were mock-infected or infected with PDCoV (MOI = 0.1). Virus-specific CPEs were observed and photographed at 12 and 24 hpi using a bright-field microscope, indicated by red arrow. (D) C16orf62 knockout LLC-PK1 cells were infected with PDCoV-GFP (MOI = 0.1) for 12 and 24 hpi, after which infected (GFP-positive) cells were visualized by fluorescence microscopy upon staining of the cell nuclei with DAPI, or (E) quantified by flow cytometry analysis. (F) At 24 hpi, RNA was extracted from supernatants and viral RNA was quantified by qRT–PCR. (G) One-step growth curves of LLC-PK1 WT and C16orf62 knockout cells infected with PDCoV (MOI = 0.1) measured by TCID_50_ assay. (H) C16orf62 knockout ST cells were infected with PDCoV-GFP (MOI = 0.1) for 12 and 24 hpi, after which infected (GFP-positive) cells were visualized by fluorescence microscopy upon staining of the cell nuclei with DAPI, or (I) quantified by flow cytometry analysis. (J) Indirect immunofluorescence analysis of the C16orf62-rescue ST cells infected with PDCoV (MOI = 0.1) for 24 h. Infected cells were stained with an anti-PDCoV S1-specific antibody (green). (K) Quantification of the fluorescence intensity from (J) by ImageJ. Nuclei were stained with DAPI (blue). Images were taken at 10× magnification. Scale bar = 200 μm. Error bars represent standard deviations from three independent experimental replicates. **p < .01, ***p < .001, ****p < .0001.

**Figure:**
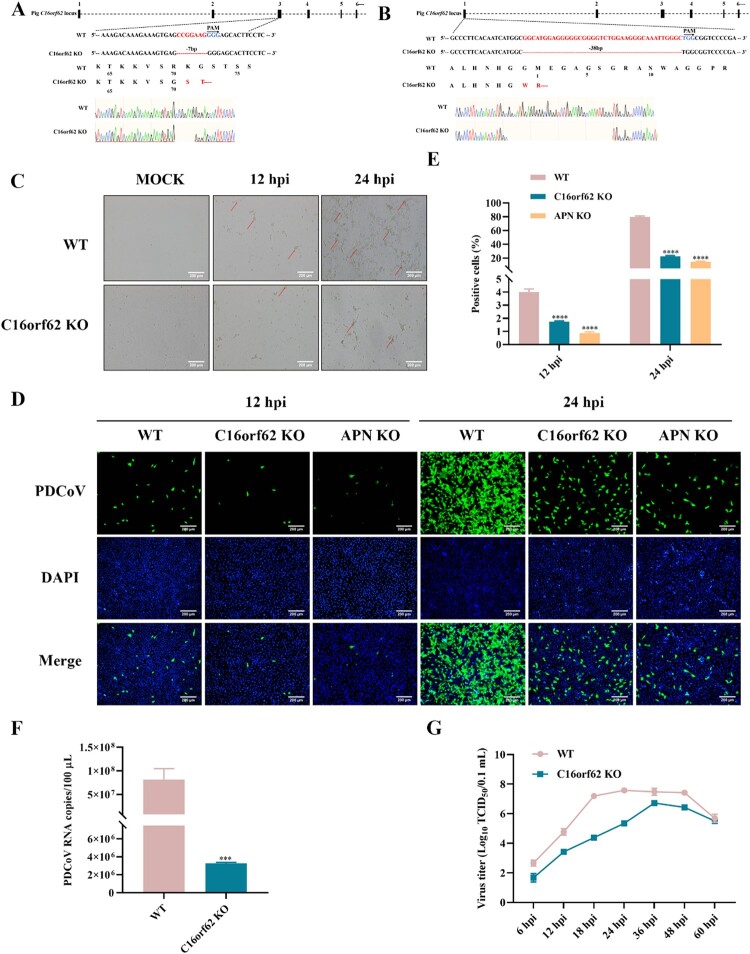

**Figure:**
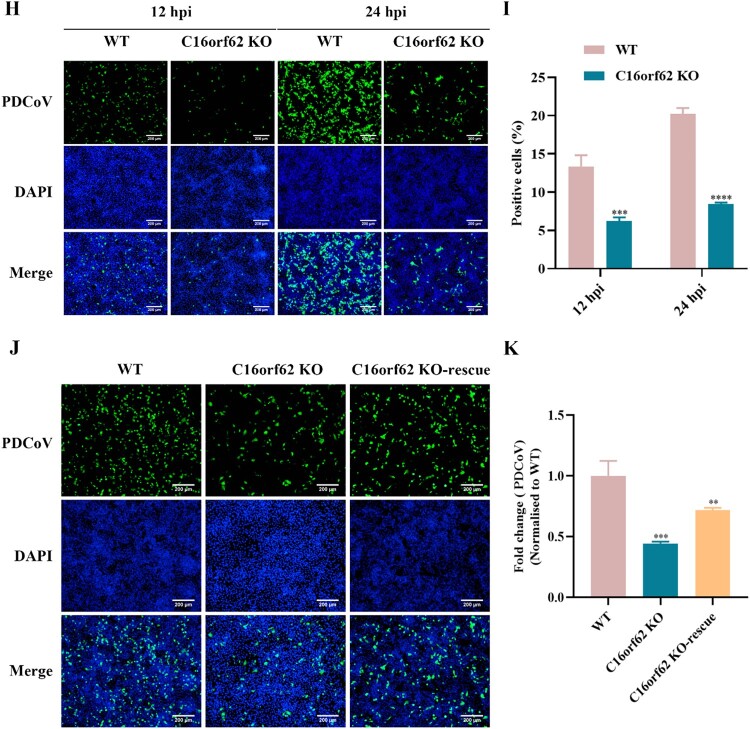

### C16orf62 overexpression enhances PDCoV infection across diverse cell lines

**Figure 4.**
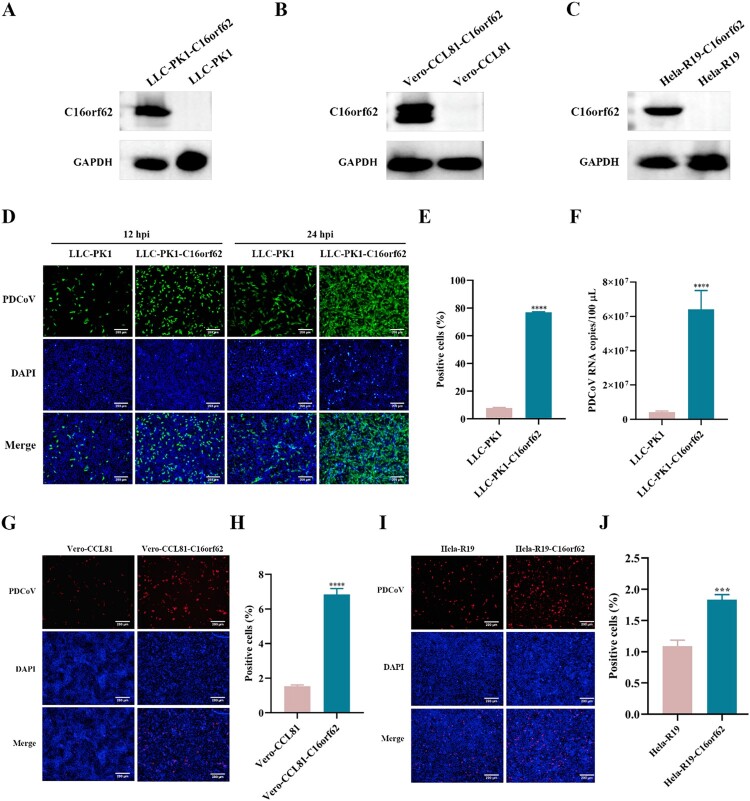
Overexpression of C16orf62 enhances PDCoV infection in multiple cell types. (A) Western blot analysis confirming the overexpression of C16orf62 in LLC-PK1 cells using an anti-V5 mouse antibody. (B) LLC-PK1 WT and C16orf62-overexpressing cells were infected with PDCoV-GFP (MOI = 0.1) for 12 and 24 hpi, after which infected (GFP-positive) cells were visualized by fluorescence microscopy upon staining of the cell nuclei with DAPI, or (C) quantified by flow cytometry analysis at 24 hpi. (D) qRT–PCR analysis of PDCoV infection (MOI = 0.1) in C16orf62-overexpressing LLC-PK1 and WT cells at 24 hpi. (E–F) Western blot analysis confirming the expression of C16orf62 in Vero-CCL81 and Hela-R19 cells using an anti-V5 mouse antibody. (G) At 24 hpi, PDCoV (MOI = 50) replication in the WT and C16orf62-overexpressing Vero-CCL81 cells was determined by IFA assay upon staining of the cell nuclei with DAPI, or (H) quantified by flow cytometry analysis. (I) At 24 hpi, PDCoV (MOI = 50) replication in the WT and C16orf62-overexpressing Hela-R19 cells was determined by IFA assay using an anti-PDCoV S1 human antibody upon staining of the cell nuclei with DAPI, or (J) quantified by flow cytometry analysis. Red, immunofluorescence signals. Scale bar = 200 μm. Error bars represent standard deviations from three independent experimental replicates. *p < .05, **p < .01, ***p < .001 and ****P <.0001.

To further investigate the role of C16orf62 in PDCoV infection, LLC-PK1 cells stably overexpressing V5-tagged C16orf62 were generated and infected with PDCoV-GFP alongside parental LLC-PK1 cells (Figure 4(A)). Overexpression of C16orf62 promoted the infection of PDCoV, as evidenced by increased GFP-positive cells observed using both microscopy and flow cytometry (Figure 4(D and E)). This finding was further supported by qRT–PCR analysis (Figure 4(F)). We next determined whether C16orf62 could indeed promote PDCoV infection in Vero-CCL81 and HeLa-R19 cells, which are poorly susceptible due to a lack of detectable APN expression [17]. Mutant Vero-CCL81 and HeLa-R19 cells stably expressing C16orf62 were generated and verified by Western blot analysis (Figure 4(B and C)). Compared to the inefficient infection observed in Vero-CCL81 and HeLa-R19 cells, overexpression of C16orf62 potentiated PDCoV infection (Figure 4(G–J)). These findings collectively demonstrate that C16orf62 is a host factor required for PDCoV infection without the need of APN expression.

### C16orf62 is required for PDCoV attachment and internalization

**Figure 5.**
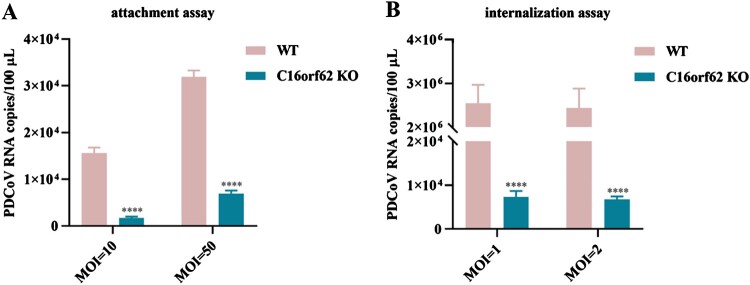
C16orf62 is required for PDCoV attachment and internalization. Viral attachment (A) and internalization (B) were assessed in WT and C16orf62 knockout LLC-PK1 cells by a qRT–PCR analysis of PDCoV M copy numbers. Error bars represent standard deviations from three independent experimental replicates. ****p < .0001.

Having confirmed the importance of C16orf62 in facilitating PDCoV infection, we next determined which stage of the viral life cycle was affected in the knockout clones. To further investigate this, we performed virus binding and internalization assays using a qRT–PCR-based method to quantify the PDCoV bound to the cells or internalized into cells. In the binding assay, C16orf62 knockout cells exhibited a significant reduction in the binding of PDCoV particles compared to WT cells (Figure 5(A)). In the internalization assay, viral internalization was also reduced in C16orf62 knockout cells (Figure 5(B)). These findings suggest that C16orf62 is essential for PDCoV attachment and internalization.

### No direct interaction between c16orf62 and PDCoV S1

**Figure 6.**
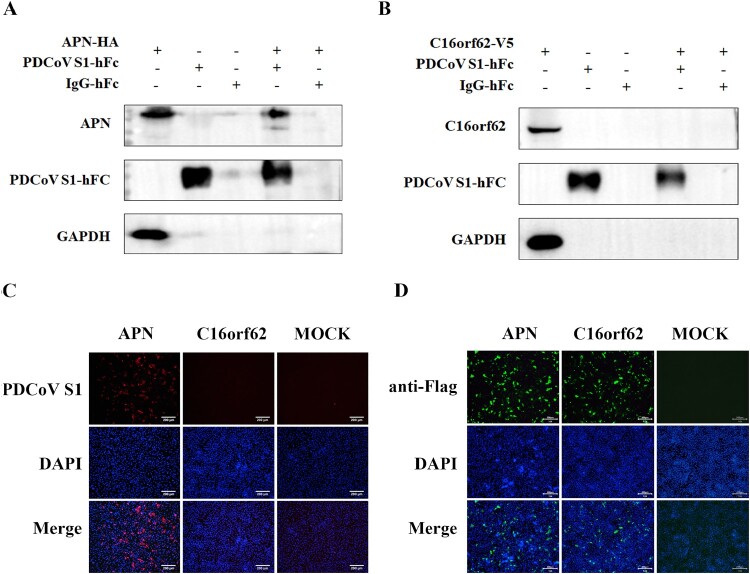
C16orf62 is not the receptor for PDCoV. (A) Co-IP analysis to assess PDCoV S1 binding to APN. 1 × 10^7^ HEK 293 T cells were seeded in 10-cm cell culture dishes and transfected with pQCXIP-APN-HA for 36 h and lysed with Cell Lysis Buffer for Western blot and IP. Subsequently, cell lysates were incubated with recombinant Fc-tagged PDCoV S1 protein, and the Fc-tagged S1-protein was subsequently precipitated by protein A-coupled agarose beads. Co-purification of APN (through binding to S1) was assessed by Western blot using an anti-APN antibody. (B) Co-IP analysis to assess PDCoV S1 binding to C16orf62. 1 × 10^7^ ST cells overexpressing C16orf62-V5 were seeded in 10-cm cell culture dishes. Subsequently, the cell lysates were incubated with recombinant Fc-tagged PDCoV S1 protein, and precipitated by protein A-coupled agarose beads, and Co-purification of C16orf62-V5 was assessed by Western blot using an anti-V5 antibody. (C) Vero-CCL81 cells were transfected with pQCXIP-APN-Flag or pQCXIP-C16orf6-Flag in 24-well plates, fixed with 4% paraformaldehyde for 15 min at room temperature and washed three times with PBS and incubated with PDCoV S1 protein (10 μg/mL) at 4°C for 2 h, and binding was detected using an Alexa 594-conjugated anti-human antibody. Blue, DAPI-stained nuclei. Red, immunofluorescence signals. (D) Verification of APN and C16orf62 overexpression in Vero-CCL81 cells. The pQCXIP-APN-Flag and pQCXIP-C16orf6-Flag were transfected into Vero-CCL81 cells in 24-well plates, and gene expression was detected by IFA using an anti-Flag antibody. Blue, DAPI-stained nuclei. Green, immunofluorescence signals. Scale bar = 200 μm.

To test the interaction of C16orf62 with the PDCoV S1, ST cells were stably expressing V5-tagged C16orf62 followed by Co-IP with soluble PDCoV S1-Fc fusion protein. Additionally, HEK 293 T cells were transfected with expression plasmids encoding HA-tagged APN to serve as a positive control [38]. The results indicated that PDCoV S1 protein was coprecipitated with APN, but not with C16orf62 (Figure 6(A and B)). To further confirm the interaction between PDCoV S1 and C16orf62, a surface binding assay was performed in Vero-CCL81-C16orf62 cells using PDCoV S1-Fc, with Vero CCL81-APN serving as a positive control [39]. The results also indicated that the soluble PDCoV-S1 protein binds to APN overexpressing Vero-CCL81 cells, but showed no detectable binding to those expressing C16orf62 (Figure 6(C and D)). Although we did not find any evidence of direct interaction between C16orf62 and the viral spike protein, the data suggests that C16orf62 may be involved in PDCoV infection through other mechanisms.

### C16orf62 depletion reduces cellular APN expression levels

**Figure 7.**
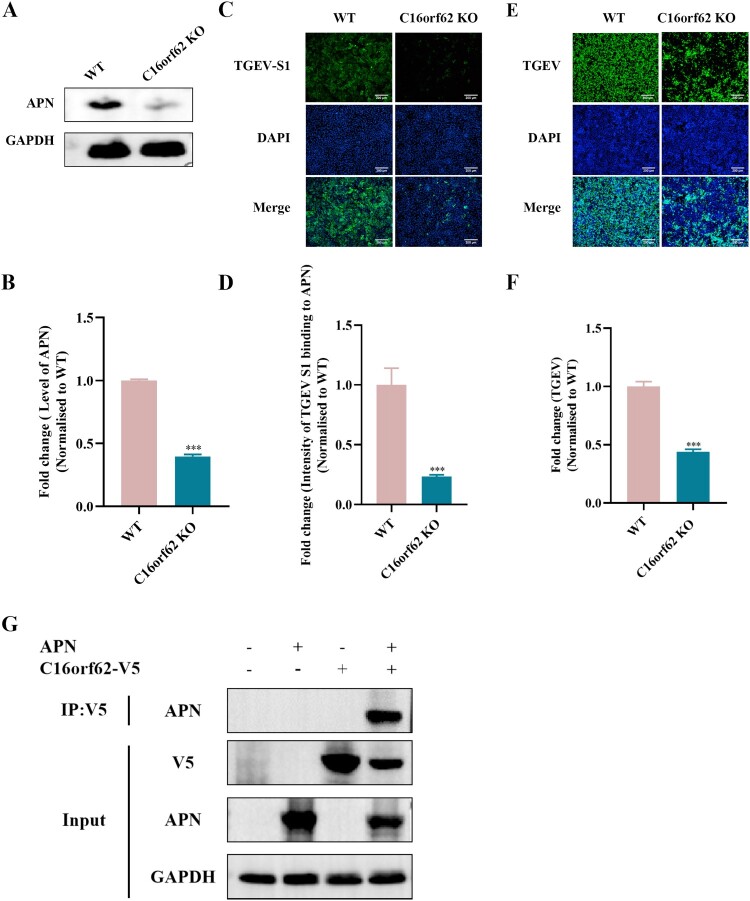
C16orf62 knockout in ST cells reduces APN expression. (A) Western blot analysis showing the protein levels of APN in C16orf62 knockout clones compared to ST cells using an anti-APN antibody. (B) Quantitative analysis of APN protein levels based on the Western blot data in (A) by ImageJ. (C) Binding of TGEV S1 protein to the cell surface APN of ST WT and C16orf62 knockout cells. (D) Quantification of the fluorescence intensity from (C) by ImageJ. (E) At 24 hpi, TGEV (MOI = 0.1) replication in the WT and C16orf62 knockout ST cells was determined by IFA assay using an anti-TGEV S1 antibody upon staining of the cell nuclei with DAPI, or (F) quantified by ImageJ. Green, immunofluorescence signals. (G) Validation of interaction between the C16orf62 and APN with Co-IP analysis. Immunoblot of C16orf62-V5 recombinant proteins from overexpressing C16orf62 ST cells using anti-APN mAb. Scale bar = 200 μm.

C16orf62 is a member of the retriever complex which is involved in regulating the retrograde transport of proteins from endosomes to the trans-Golgi network (TGN) or the plasma membrane. This transport process is crucial for maintaining proper cellular function, including the sorting and recycling of membrane proteins, such as receptors and transporters [40–42]. Since the PDCoV receptor APN is being endocytosed, we hypothesized that C16orf62 could promote the retrograde transport of internalized APN from endosomes to cell surface. To test this, we detected the effect of C16orf62 knockout on APN protein expression using Western blot by anti-APN antibody. The results showed that the expression of receptor APN was decreased in C16orf62 knockout ST cells (Figure 7(A and B)). Furthermore, we performed a binding assay using the soluble TGEV S1 protein, which is known to interact with APN on the cell surface [39]. The result indicated that the binding signal of TGEV-S1 to knockout cells were significantly reduced compared to WT cells (Figure 7(C and D)). We also found that knocking out C16orf62 inhibited TGEV infection (Figure 7(E and F)), consistent with the downregulation of APN expression. To directly test the interaction between C16orf62 and APN, Co-IP was performed with anti-APN mAb to capture protein complexes. The results showed a significant interaction between C16orf62 and APN (Figure 7(G)). To further confirm the interaction of C16orf62 and APN, the confocal microscopy experiment was conducted. There was an obvious colocalization between C16orf62 and APN (Figure S3). These results highlight the interaction between C16orf62 and APN and support the notion that the reduced PDCoV infection observed in C16orf62 knockout cells is associated with the decreased APN expression.

## Discussion

PDCoV, an emerging enteropathogenic swine virus, poses a severe threat to human and animal health worldwide [23,43]. Unfortunately, little is known regarding its pathogenesis and virus-host interactions, and there are no effective drugs or vaccines to control the disease [44]. Although several host factors, including APN [17,45], TMEM41B [24], SLC35A1 [23], CTSL and CTSB [45], have been reported to be involved in PDCoV infection, the entry mechanism of PDCoV remains largely unclear [25,46]. Genome-wide CRISPR/Cas9 library screening has proven to be a valuable tool to study gene function, enabling an unbiased interrogation of gene function in a wide range of species, particularly in the identification of host factors for various pathogens, such as SARS-CoV-2 [34], influenza A virus (IAV) and Japanese encephalitis virus (JEV) [47].

In this study, by performing a genome-wide CRISPR-Cas9 knockout screen, we revealed C16orf62 as a host factor required for PDCoV infection that acts by regulating cell-surface APN, which has been reported as the protein receptor for PDCoV [17,48]. Our screening results also identified the ANPEP gene as a top hit, which was previously reported as the main functional receptor for several coronaviruses, including transmissible gastroenteritis virus (TGEV), human coronavirus 229E (HCoV-229E), and type II feline coronavirus (FCoV) [49–51], validating the effectiveness and reliability of our method. The ability of C16orf62 knockout cells to inhibit PDCoV replication was comparable to that observed with APN knockout cells. PDCoV infection in porcine cells was significantly reduced in C16orf62 knockout cells, and this reduction could be rescued by reconstituting C16orf62 expression. Notably, PDCoV efficiently infects cells of various species that overexpress C16orf62, including cells with human, monkey and pig origin. These findings emphasize the broad species range of PDCoV across different hosts, and highlight the critical role of C16orf62 in PDCoV infection process. Additionally, we determined that C16orf62 is critical for the adsorption and internalization of PDCoV. The binding of cell surface receptors to ligands initiates endocytosis, and the fate of endocytosed receptors and complexes is determined by transport complexes such as the retromer, also known as the reverse vesicle transport complex [52,53]. This is a crucial process in viral invasion, infection, and replication [54,55]. C16orf62 is a vital component of the retriever complex, a multi-protein structure that shares both structural and functional similarities with retriever. This retriever complex, consisting of DSCR3, C16orf62, and VPS29, is primarily localized within endosomes [56], with DSCR3 and VPS29 both found as enriched hits in the screening, yet ranking lower compared to C16orf62. C16orf62 interacts with the cargo adaptor SNX17 (and SNX31), facilitating the retrieval and recycling of specific cargo proteins from endosomes to TGN or the plasma membrane for reuse, thereby influencing various cellular process [57]. Here, we speculate that the receptor of PDCoV, APN, acts as cargo for the retriever complex and is regulated by C16orf62. Following the knockout of C16orf62, we assessed the expression of APN. The results showed that the expression of receptor APN was decreased in C16orf62 knockout cells, accompanied by a significant reduction in its binding signal of TGEV-S1. We also tested TGEV infection in ST C16orf62 knockout cells, which further validated the fact that APN was downregulated. Additionally, their interaction was confirmed by Co-IP assay and confocal microscopy. However, the exact molecular mechanisms by which C16orf62 regulates APN action remain unclear and require further investigations. Taken together, these data suggest that the potential of C16orf62 as a key host factor for PDCoV infection, and this could serve as antiviral drug target for PDCoV. Additionally, overexpression of C16orf62 in Vero-CCL81 and HeLa-R19 cells, which are poorly susceptible to PDCoV infection due to a lack of detectable APN expression, also enhanced PDCoV infection. This suggests that C16orf62 has another effect in promoting PDCoV infection, other than regulating APN expression.

In addition to C16orf62, a number of other candidate host factors involved in PDCoV infection, including FAM150A, were identified in our CRISPR screen, warranting further investigation. Anaplastic lymphoma kinase (ALK) and the related leukocyte tyrosine kinase (LTK) are tyrosine kinases that have recently been characterized. They are activated by their ligands, ALKAL1 and ALKAL2 (also called FAM150A and FAM150B or AUGβ and AUGα, respectively), and are involved in neural development, cancer and autoimmune diseases [58]. However, the specific mechanisms by which these host proteins affect viral infection requires further investigation.

In summary, a genome-wide CRISPR/Cas9 library knockout screen conducted using PDCoV in human cells not only confirmed APN as a receptor for PDCoV infection, but also demonstrated C16orf62 as a pivotal host factor for PDCoV infection that acts by regulating APN. This is the first time C16orf62’s role in coronavirus infection has been elucidated. Our study emphasizes the crucial role of C16orf62 in coronavirus infection, highlighting its potential as a target for the development of anti-coronavirus therapeutics.

## Supplementary Material

Supplemental Material

Supplemental Material

Supplemental Material

Supplemental Material
