# Supplementary figures and images for: Genome-wide CRISPR/Cas9 library screen identifies C16orf62 as a host dependency factor for porcine deltacoronavirus infection
Source: Emerg Microbes Infect. 2024 Sep 2;13(1):2400559. doi: 10.1080/22221751.2024.2400559 (PMC11404382; doi:10.1080/22221751.2024.2400559)

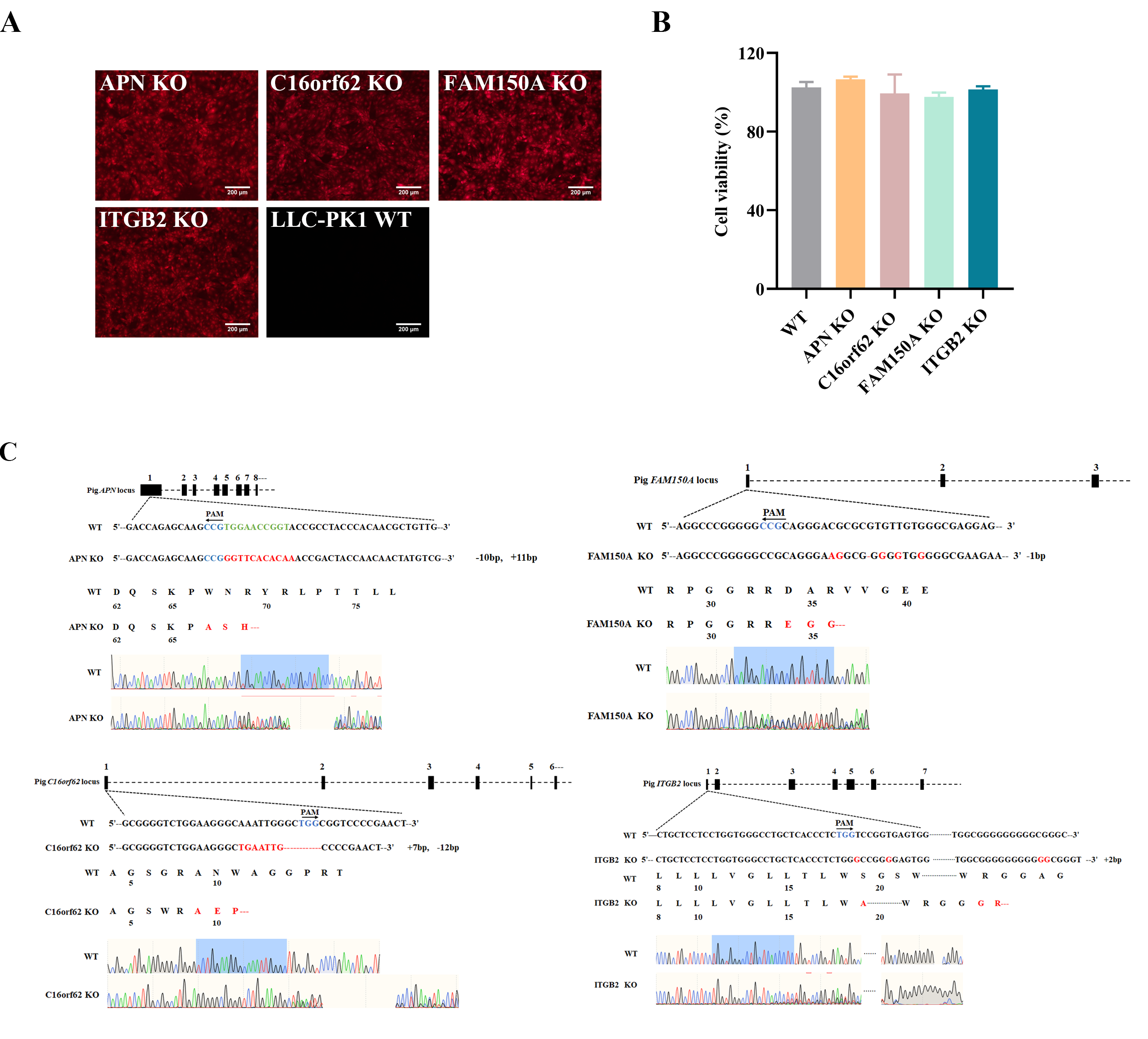

Supplement: Supplemental Material [file TEMI_A_2400559_SM8174.tif]

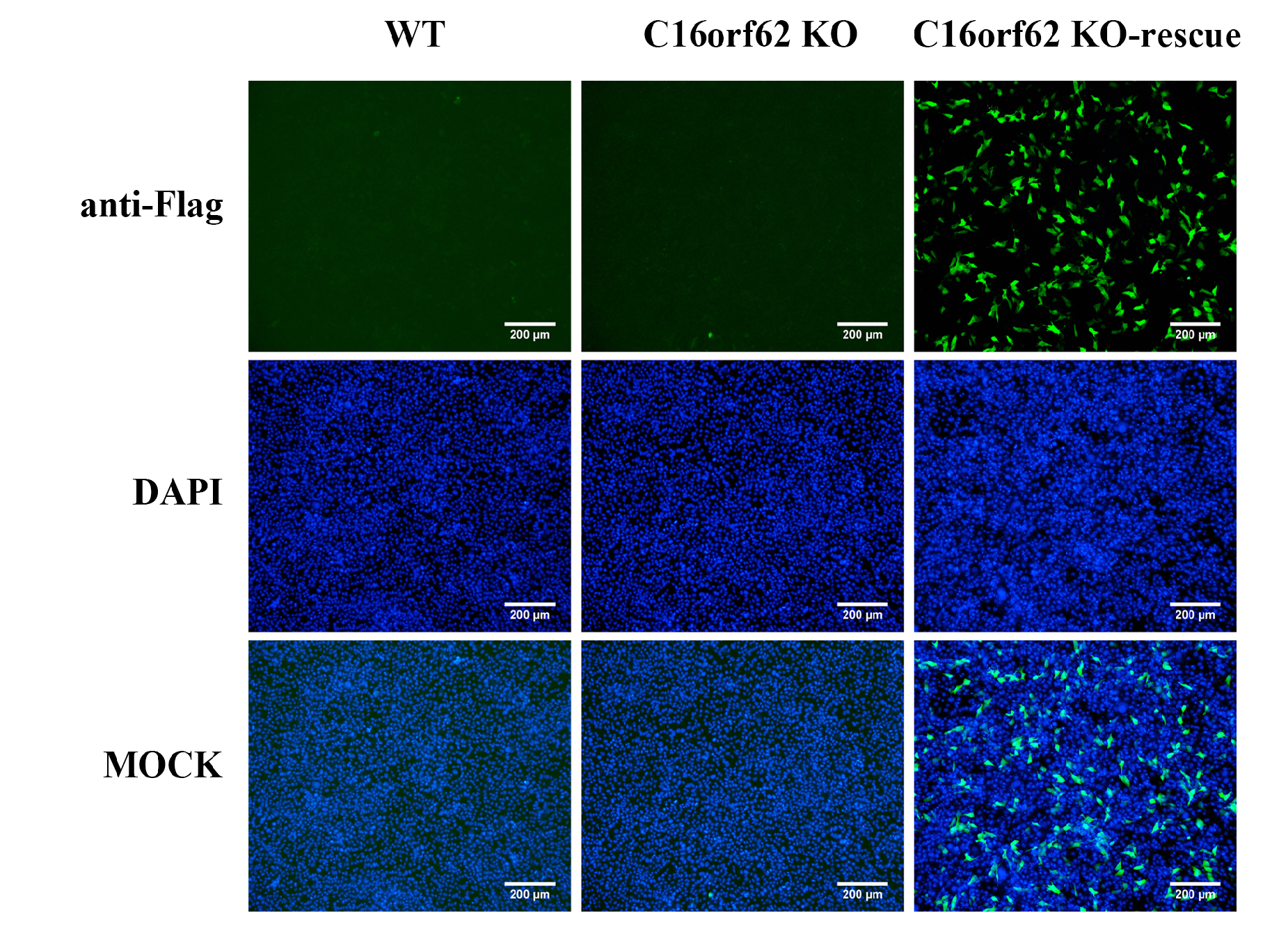

Supplement: Supplemental Material [file TEMI_A_2400559_SM8169.tif]

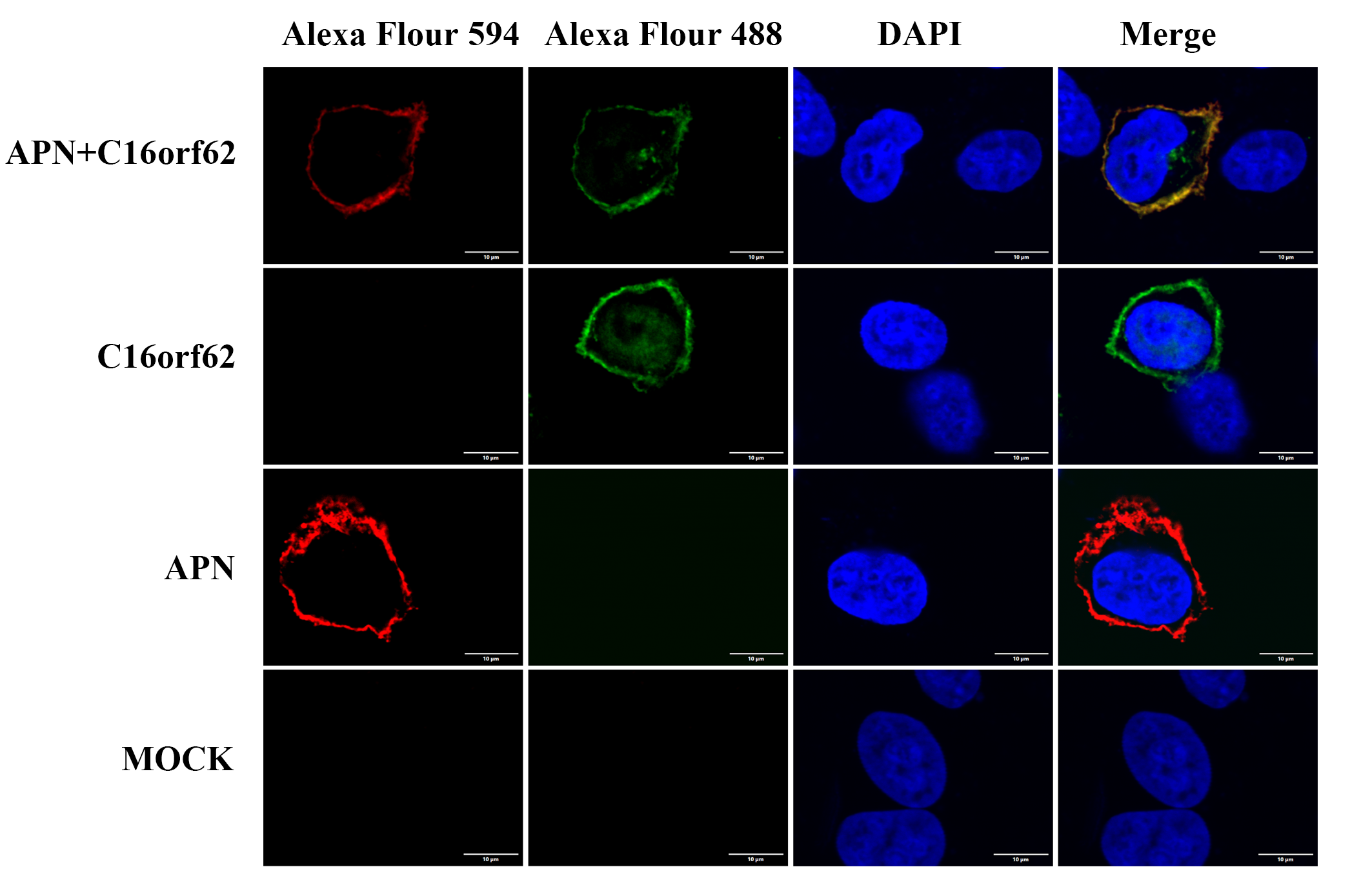

Supplement: Supplemental Material [file TEMI_A_2400559_SM8168.tif]
